# A Single Plasmid of Nisin-Controlled Bovine and Human Lactoferrin Expressing Elevated Antibacterial Activity of Lactoferrin-Resistant Probiotic Strains

**DOI:** 10.3390/antibiotics10020120

**Published:** 2021-01-27

**Authors:** Zhen-Shu Liu, Chuen-Fu Lin, Chung-Pei Lee, Min-Chi Hsieh, Hung-Fu Lu, Ying-Fang Chen, Yu-We Ku, Po-Wen Chen

**Affiliations:** 1 Department of Safety, Health and Environmental Engineering, Ming Chi University of Technology, New Taipei City 24301, Taiwan; zsliu@mail.mcut.edu.tw (Z.-S.L.); alice810323@gmail.com (M.-C.H.); frank569497975@gmail.com (H.-F.L.); 2 Chronic Diseases and Health Promotion Research Center, Chang Gung University of Science and Technology, Chiayi 61363, Taiwan; 3 Center for Environmental Sustainability and Human Health, Ming Chi University of Technology, New Taipei City, 24301, Taiwan; 4 Department of Veterinary Medicine, College of Veterinary Medicine, National Pingtung University of Science and Technology, Pingtung 91201, Taiwan; cflin2283@mail.npust.edu.tw; 5 School of Nursing, National Taipei University of Nursing and Health Sciences, Taipei 11219, Taiwan; chungpei@ntunhs.edu.tw; 6 Department of Veterinary Medicine, National Chung Hsing University, Taichung 40249, Taiwan; taylorswiftellen@gmail.com (Y.-F.C.); kyw168@mail.e-land.gov.tw (Y.-W.K.)

**Keywords:** recombinant lactoferrin, probiotic, antibacterial activity, food-borne pathogen

## Abstract

Lactoferrin (LF) is a multifunctional protein found in mammals, and it shows broad-spectrum antimicrobial activity. To improve the functional properties of specific probiotics in order to provide both the beneficial characteristics of lactic acid bacteria and the biological activity of LF, cDNAs of bovine LF (BLF), human LF (HLF), or porcine LF (PLF) were cloned into a nisin-inducible plasmid. These were then transformed into the selected eight probiotics, which are LF-resistant hosts. Expression of recombinant LFs (rLFs) was analyzed via SDS-PAGE and Western blot analysis. Although the selected host strains may not contain the *nisRK* genes (NisK, the sensor kinase; NisR, the regulator protein), the components of autoregulation, a low level of LFs expression can be successfully induced by using nisin within bacterial cells in a time-dependent manner in three engineered clones, including *Lactobacillus delbrueckii*/HLF, *L. delbrueckii*/BLF, and *L. gasseri*/BLF. *Lactobacillus delbrueckii* and *Lactobacillus gasseri* originate from yogurt and human milk, respectively, and both strains are functional probiotic strains. Therefore, we further compared the antibacterial activities of disrupted recombinant probiotic clones, conventional strains (host control), and vector control ones by using agar diffusion and broth inhibition analysis, and the expression of rLFs in the above three clones considerately improved their antibacterial efficacies against four important food-borne pathogens, namely, *Escherichia coli*, *Staphylococcus aureus*, *Enterococcus faecalis*, and *Salmonella* *enterica*. In conclusion, this study provides a simple strategy for the production of functional LFs (BLF and HLF) in both functional and LF-resistant hosts for applications in the field.

## 1. Introduction

Lactoferrin (LF) is an 80 kDa iron-binding protein present mostly in the milk and exocrine fluids of mammals, and it has been shown to display extensive biological effects, including antimicrobial, anti-inflammatory, and immune-modulating properties. Thus, the application of LF to various fields has been continuously studied [1,2,3]. Notably, although LF possesses antimicrobial activity against a wide spectrum of pathogens, it exerts minor antibacterial activities on specific probiotic strains, and it even possesses prebiotic activity on specific probiotic strains [4,5,6]. Because of the roles of LF in mediating many physiological functions, it has been recommended as a good food additive or biotherapeutic agent. For example, there is evidence indicating that oral LF could be effective in the prevention of sepsis and necrotizing enterocolitis in preterm neonates, although this is not yet fully established [7]. Moreover, in animal models, LF supplementation is effective in preventing preterm delivery and intrauterine infections [8,9]. Furthermore, early-life LF administration can enhance growth performance and decrease diarrhea incidence in suckling piglets by promoting the development of intestinal functions and by modifying the microbiota in the small intestine [10]. Additionally, the simultaneous combination of LF preparations and specific lactobacilli strains can improve women’s health through probiotic and prebiotic input [11].

As described above, LF is a multifunctional protein that has been used in the control of various diseases. Since purified mammalian LFs are quite expensive, some studies have attempted to produce LF using various systems, for which probiotic systems have also been employed. For reference, *Lactobacillus casei* has been used to express human LF (HLF), and the *L. casei/*HLF strain generated was shown to display antibacterial activity both in vitro and in vivo, where it served to protect mice against *Escherichia coli* infection [12]. Moreover, *L. casei* was further used to express bovine LF (BLF), and the *L. casei*/BLF strain displayed antifungal activity against vulvovaginal candidiasis in a mouse model [13]. In addition, *Lactobacillus pentosus* has been used to express porcine LF (PLF), and *L. pentosus/*PLF supplementation has been described to elevate antibacterial activity and improve the efficacy of vaccination against Aujeszky’s disease in a mouse model. This report also suggests that recombinant *L. pentosus* could provide both the beneficial characteristics of lactic acid bacteria and the biological activity of lactoferrin [14]. Finally, *L. plantarum* has been employed for expression of PLF and was used as a feed additive in the daily diet of weaned piglets, and addition of recombinant *L. plantarum*/PLF led to a significant increase in average daily gain and feed intake, improved feed efficiency, and reduced incidence of diarrhea in these piglets [15]. Collectively, these previous reports support the possibility of using specific probiotic strains for the production of functional LFs.

Nisin is a highly stable post-translationally modified antimicrobial peptide that is secreted by *Lactococcus lactis*. It displays strong antimicrobial activity against a wide range of Gram-positive organisms. Thus, it has been widely applied in both the food and medical arenas [16,17,18]. Moreover, a gene expression system has been developed due to the autoregulatory properties of the *L. lactis* nisin gene cluster, and two of the clustered genes, nisA and NisF, can be induced by nisin through a two-component signal transduction pathway, which consists of a histidine protein kinase, NisK, and a response regulator, NisR; the expression of nisR and nisK is driven from the constitutive promoter of nisR. Collectively, in this kind of nisin-inducible expression, a specific host strain which harbors nisK and nisR should be used, as previous reports indicate that the nisRK genes are the only nis genes required for nisin-mediated signal transduction and nisA or nisF promoter activation in *Lactococcus lactis* [19,20]. However, induction of the nisA promoter can also occur when introduced into the plasmid-free *L. lactis* during growth in galactose or lactose, independent of the NisRK system [21]. In addition, a transferable dual-plasmid inducible gene expression system has been developed and functionally implemented in lactic acid bacteria other than *Lactococcus lactis* [22]. Collectively, in the present work, we tried to test the possibility about applying the nisin-control gene expression (NICE) system to express rLFs in specific hosts using only one plasmid as described below.

We previously demonstrated that the combination of natural BLF and specific probiotics can lead to synergistic antibacterial activity in vitro [23,24]. Furthermore, we also showed that BLF can enhance the growth of specific probiotic strains in a dose-dependent manner [4]. Therefore, in the present study, we tried to confer the prebiotic properties of LF on specific probiotic strains, and probiotic strains that displayed enhanced growth rates in the presence of bovine lactoferrin or those that could withstand the antibacterial activity of bovine lactoferrin were used as host-friendly bioreactors to produce recombinant bovine, human, or porcine lactoferrin by introducing lactoferrin expression plasmids (pNZ8148/LFs: nisin-inducible promoter) into these probiotics. Finally, we obtained a series of probiotic candidates that could potentially express BLF, HLF, or PLF in different hosts. We initially demonstrated that at least three recombinant probiotic clones of two functional hosts can produce low level but functional LFs to strongly elevate the antibacterial activity of LF-resistant hosts against four important food-borne pathogens. Therefore, this study provides a simple strategy for the production of recombinant LFs in functional hosts for applications in the field.

## 2. Materials and Methods

### 2.1. Bacterial Strains and Growth Conditions

The probiotic bacterial strains used in this study, including *Lactobacillus delbrueckii* (BCRC 14008), *Lactiplantibacillus paraplantarum* (old species name: *Lactobacillus paraplantarum*; ATCC 700210) [25], *Lactobacillus gasseri* (laboratory stock, isolated from human milk), *Lacticaseibacillus rhamnosus* (old species names: *Lactobacillus rhamnosus* (ATCC 53103) [25], *Pediococcus pentosaceus* (ATCC 8081), *Bifidobacterium angulatum* (ATCC 27535), *Bifidobacterium breve* (BCRC1258), and *Bifidobacterium catenulatum* (ATCC 27539), were cultured in deMan–Rogosa–Sharpe (MRS) medium (Oxoid) at 37 °C without agitation. *E. coli* TG1 (laboratory stock) was cultured in Luria–Bertani medium at 37 °C with agitation. When required, various concentrations of chloramphenicol (ranging from 2.5 to 20 μg/mL) were added to *E. coli* TG1 and probiotic cultures, serving as selection markers. For analyzing antibacterial activities, food-borne pathogenic strains were acquired, including *E. coli* (HER1255), *Enterococcus faecalis* (ATCC 29212), *Staphylococcus aureus* (ATCC 25923), and *Salmonella typhimurium* (ATCC 14028). These pathogenic strains were cultured in tryptic soy broth (TSB) at 37 °C under aerobic conditions.

### 2.2. Cloning of BLF and HLF Genes into Shuttle Plasmids

Molecular manipulations, including polymerase chain reaction (PCR), genome and plasmid isolations, electrophoresis, restriction endonuclease digestion, and fragment ligation, were conducted according to standard techniques [26]. The primers used in this study are as follows: BLF-1 GCTCTAGAATGAAGCTCTTCGTCCCCG (contains *XbaI* restriction site) and BLF-2 CGAGCTCTTACCTCGTCAGGAAGGCG (contains *SacI* restriction site); HLF-1 GGGGTACCATGAAACTTGTCTTCCTCGTC (contains *KpnI* restriction site) and HLF-2 CCCAAGCTTTTACTTCCTGAGGAATTCACAG (contains *HindIII* restriction site); PLF-1 GGGGTACCATGAAGCTCTTCATCCCCGC (contains *KpnI* restriction site) and PLF-2 CCCAAGCTTTCAGGTAGCGATGGCTGTGA(contains *HindIII*restriction site). BLF cDNA (2.2 kb) was amplified from a cDNA clone (Clone ID: BC116051, Transomic), and subsequently cloned into the NICE vector pNZ8148 (Cmp^r^, nisin-inducible promoter), resulting in the pNZ8148/BLF plasmid. HLF cDNA (2.1 kb) was amplified from a cDNA clone (Clone ID: CH848574, Transomic), and subsequently cloned into the NICE vector pNZ8148, resulting in the pNZ8148/HLF plasmid. Porcine LF cDNA (2.0-kb) was amplified from pBS-PLF kindly provided by Dr. Chen, Chuan-Mu (National Chung Hsing University), and was also sub-cloned into NICE vector pNZ8148, resulting in plasmid pNZ8148/PLF. The constructs obtained were analyzed by nucleotide sequencing and subjected to bacterial transformation and electroporation, as described below.

### 2.3. Bacterial Transformation and Electroporation

To obtain sufficient quantities of the constructed plasmids (pNZ8148/BLF, pNZ8148/HLF, and pNZ8148/PLF) for electroporation, they were introduced into *E. coli* TG1 using the CaCl_2_ method in combination with the heat shock approach [26]. Then, a large quantity of plasmid DNA was extracted using the Viogene DNA extraction kit. For the preparation of electrocompetent cells, probiotic strains were grown in MRS broth at 37 °C to an optimal optical density at 600 nm (OD_600_) of 0.3 to 0.5, after which the bacteria were ice bathed for at least 10 min. Bacterial cells were harvested by centrifugation (5000× *g*, 15 min, 4 °C), and subsequently washed three times with cold distilled water. Finally, the cells were resuspended in 100 μL of 10% glycerol. Electroporation was carried out by mixing 100 μL of resuspended cells with 0.3 to1 μg of plasmid DNA. The suspension was transferred to a disposable cuvette (Bio-Rad Laboratory, Richmond, CA, USA) with a 0.2 cm electrode gap and subjected to an electric pulse using a MicroPulser (Bio-Rad Laboratory, Richmond, CA, USA). Transformed cells were diluted in 1 mL of recovery medium and incubated at 37 °C for 3 h. Finally, transformed cells were cultured on MRS plates or in MRS broth supplemented with 2.5 to 20 µg/mL chloramphenicol for the selection of transformants.

### 2.4. Analysis of LF Expression in Various Probiotic Transformants

Various transformed probiotic clones were cultured in MRS broth, and expression of HLF or BLF was induced under different conditions: LF expression was induced by the addition of 0.1 to 20 ng/mL of nisin for 0 to 8 h. The cell pellets were lysed with an ultrasonic cell disruptor (Sonics & Materials, VCX 600, Newtown, CT, USA) on ice, and cell lysates were analyzed via SDS-PAGE and Western blot analysis. The nitrocellulose membrane for Western blot analysis was incubated with rabbit anti-HLF primary antibody (1:20,000 dilution; Upstate, Cat: 07-685) and subsequently with horseradish peroxidase-conjugated goat anti-rabbit IgG secondary antibody (1:3000 dilution; Invitrogen, Cat: 65-6120).

### 2.5. Determination of LF Concentration in Probiotic Transformants

To evaluate the level of recombinant LF expression in recombinant probiotic clone, *L. gasseri*/BLF was grown in 10 mL MRS broth at 37 °C to an optimal OD_600_ of 1.2. LF expression was then induced by adding nisin at a concentration of 1 ng/mL (supplemented in fresh medium) for 2, 4 or 6 h at 30 °C, and 3 mL of induced bacterial culture was harvested during each time intervals. Cell pellets were resuspended in about 200 μL of ice-cold Tris-HCl (1 M, pH 7.2, supplemented with 1 mM phenylmethylsulfonyl fluoride, PMSF) and were lysed with an ultrasonic cell disruptor. Thereafter, 10 μL of the obtained cell lysates and two protein standards (1, 2, and 3 μg of purified LF or bovine serum albumin) were analyzed via SDS-PAGE analysis. The densities for the expressed LFs after 2, 4, and 6 h induction were quantified using ImageQuant 5.1(GE Healthcare, Waukesha, WI, USA), and the level of LFs expression in *L. gasseri* was determined and calculated by comparison with the protein standards.

### 2.6. Antibacterial Activities of rLFs

To evaluate the recombinant probiotic strains in vitro inhibition of food-borne pathogens, agar well diffusion assay was conducted according to the method of Shim, et al. and Tsai et al. with modifications [27,28]. Initially, each of three recombinant probiotic strains, i.e., *L. gasseri*/BLF, *L. delbrueckii/*HLF, and *L. delbrueckii*/BLF were grown in 48 mL MRS broth at 37 °C to an optimal OD_600_ of 1.2. Cultures were further divided into two 24 mL samples. LF expression was induced by adding nisin at a concentration of 1 ng/mL (supplemented in fresh medium) to one 24 mL culture for 16 h at 30 °C. As for the non-induced control (another 24 mL culture), nisin was not added to the medium, and these samples were also propagated for 16 h. Next, bacterial cells were harvested by centrifugation at 9000*× g* for 5 min at room temperature and washed twice using 4 mL PBS. Cells were resuspended in 1.2 mL medium and ruptured using five-second pulses with intervening five-second pauses on the ice at about 22 kHz for 80 cycles (HOYU, Ultrasonic 250, Taiwan). Then, 0.5 mL cell lysates were subjected to centrifugation at 9000*× g* for 5 min at 4 °C. Finally, we obtained 0.5 mL of cleared supernatants. Furthermore, the pathogenic bacterial broth of *E. coli* (HER 1255), *S. aureus* (ATCC 25923), *En. faecalis* (ATCC 29212), and *S. enterica* (ATCC 14028) was adjusted to an optimal OD_600_ of 0.9, and they were seeded into the MRS agar plate. Then, a 9 mm diameter hole was cut using a sterilized tip. Blank control (MRS, 110 μL) and aliquots of cleared supernatants prepared from probiotic clones (110 μL) were injected into agar wells. The plates were incubated at 37 °C for 18–24 h. The diameter of the inhibitory zone (mm) around each well was then measured. Three independent experiments were conducted, and each was performed in triplicate.

In addition, another in vitro broth inhibition analysis of antibacterial activity was conducted and modified from Woodma et al. [29]. Initially, about 8 mL of transformed probiotic cultures, i.e., *L. gasseri/*pNZ8148 (vector control), *L. gasseri*/BLF, *L. delbrueckii/*pNZ8148 (vector control), *L. delbrueckii/*HLF, and *L. delbrueckii*/BLF, was grown in MRS broth (plus cysteine if needed) for 48 h and further sub-cultured in 100 mL of fresh MRS broth at 37 °C to an optimal OD_600_ of 0.4 to 0.6. LF expression was then induced by adding nisin at a concentration of 1 ng/mL, and the cells were propagated and induced for 5 h at 37 °C. Next, the cells were harvested by centrifugation at 4000× *g* at 4 °C for 10 min and washed twice with sterile PBS. Cell pellets were resuspended in 10 mL of ice-cold Tris-HCl (1 M, pH 7.2, supplemented with 1 mM PMSF), and sonicated using five-second pulses with intervening ten-second pauses on ice for 20 min (Sonics & Materials, VCX 600, Newtown, CT, USA). Cleared supernatants were harvested after centrifugation at 10,000*× g* for 10 min at 4 °C. Pathogenic *E. coli* (HER 1255), *S. aureus* (ATCC 25923), *En. faecalis* (ATCC 29212), and *S. enterica* (ATCC 14028) were first activated and incubated in tryptic soy broth (TSB) and then washed twice by centrifugation at 4000*× g* in sterile PBS. Broths containing pathogenic bacteria were adjusted to 1 × 10^4^ CFU/mL. Then, 300 μL of each pathogenic bacterial culture was mixed with 200 μL of one of the cleared supernatants prepared from probiotic clones in a 1.5 mL Eppendorf tube. These mixtures were incubated for 24 h at 37 °C, and 200 μL of each mixture was plated onto nutrient agar (NA) plates to enumerate or reveal the remaining growth of the pathogenic bacteria. The effect of recombinant LF lysates on pathogenic bacterial growth was evaluated by comparing the remaining live bacteria counts to negative controls and lysates of non-transformed probiotic strains. Moreover, for the positive control, 12.5 μg/mL of chloramphenicol (final concentration) was added instead of the disrupted probiotic solution.

### 2.7. Recombinant Lactobacilli Growth Assay

To evaluate the effects of rLF on the growth of recombinant *Lactobacilli*, a spectrophotometric turbidity bioassay was performed as described previously [4]. Initially, *L. gasseri*/BLF, *L. delbrueckii/*HLF, and *L. delbrueckii*/BLF were first activated in MRS broth for 48 h at 37 °C. Then, each of three recombinant probiotic strains were grown in 40 mL MRS broth at 37 °C to an optimal OD_600_ of 0.2. Cultures were further divided into two 20 mL samples. LF expression was induced by adding nisin at a concentration of 1 ng/mL (supplemented in fresh medium) to one 20 mL culture at room temperature (approximately 23–25 °C). As for the non-induced control (another 20 mL culture), nisin was not added to the medium. The growth responses of each probiotic strain were measured by determining the OD_600_ of 1 mL bacterial broth at different time intervals at room temperature or 37 °C by consulting to our previous report [4]. These results are expressed as mean ± standard deviation and variations in growth curves of probiotic strains with and without rLF expression. The experiments were performed in triplicate, and the representative results are presented.

### 2.8. Statistical Analysis

Differences in the diameter of the inhibitory zone were determined using Student’s *t*-test. *P* < 0.01 or *P* < 0.001 was considered statistically significant.

## 3. Results and Discussion

In this study, to improve the functional properties of probiotic strains to provide both the beneficial characteristics of lactic acid bacteria and the biological activity of LF, full-length cDNAs of bovine, human, and porcine LFs were cloned into a highly efficient and stable expression vector. Then, we decided to choose specific host strains with several characteristics. Firstly, specific probiotic strains should display enhanced growth rates in the presence of bovine lactoferrin or withstand the antibacterial activity of bovine lactoferrin. Second, these host strains should have been reported to be functional probiotic strains or originate from natural and safe sources. Then, the selected probiotic strains were used as host-friendly bioreactors to produce recombinant bovine, human and porcine lactoferrin by introducing lactoferrin expression plasmids into these probiotics.

At present, various bio-engineered probiotic strains have been developed to improve or enhance the functional properties of conventional probiotic strains. However, there are some safety issues and concerns, because these bio-engineered probiotics are classified as genetically modified organisms (GMOs). For example, there are some concerns about the release of GMOs into the natural environment, which may contribute to the transfer of resistance genes or other genetic material to other organisms [30,31]. Thus, several strategies have been developed previously to reduce safety concerns regarding the use of GMOs, including the use of biological containment systems or thymidine-deficient strains or control of the chromosomal location of the gene [30,32]. However, the use of GMOs is still met with skepticism by the public. In the present study, pNZ8148 was selected, because it is known to be a highly efficient and stable expression vector. However, it does not meet the standards for food-grade application, as it encodes a chloramphenicol resistance gene. Finally, by taking the above issues into account, we focused on using disrupted probiotic lysates containing rLFs or other functional proteins for further applications. We believe that the use of cell lysates instead of GMO probiotics might be better accepted by the public.

### 3.1. Construction of Recombinant Vectors

**Figure 1:**
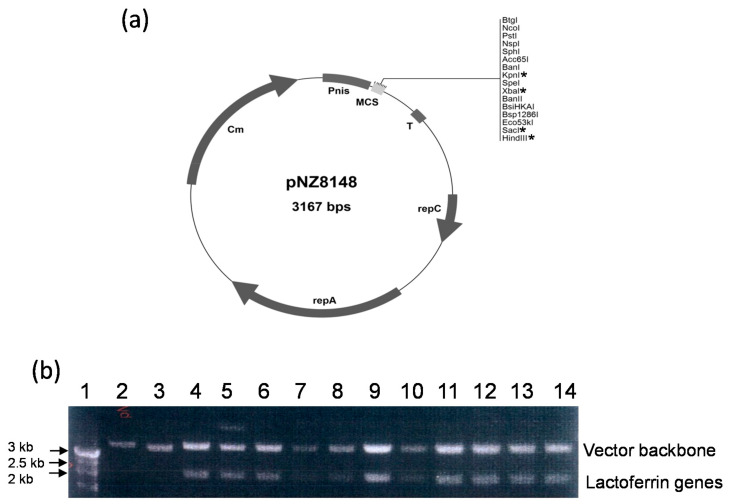
Analysis of DNA construct of pNZ-8148 and lactoferrin genes. (**a**) Vector mapping and multicloning sites. * indicates the used restriction sites in this work. (**b**) Visualization of plasmid vector and target lactoferrin genes using electrophoresis. Lane 1: marker; lane 2: pNZ8148; lane 3 to 6: amplified human lactoferrin genes; lane 7 to 10: amplified bovine lactoferrin genes; lane 11 to 14: amplified porcine lactoferrin genes.

The vector constructed in this study is shown in Figure 1. Full-length lactoferrin genes were amplified from cDNA clones (HLF and BLF genes) or the plasmid (PLF genes, from pBS-PLF) using the primers as described in the Materials and Methods Section. As highlighted in Figure 1, the three amplified genes, HLF, BLF and PLF, were inserted into the multiple cloning site of the pNZ8148 expression vector to create pNZ8148-HLF, pNZ8148-BLF, and pNZ8148-PLF, respectively. The identity of the obtained constructs was verified by nucleotide sequencing.

The human lactoferrin (HLF) cDNA fragment was PCR amplified and cloned into the *Kpn*I and *Hind*III sites of the pNZ8148 vector under the control of the nisin promoter; the bovine LF (BLF) cDNA fragment was PCR amplified and cloned into the *Xba*I and *Sac*I sites of the pNZ8148 vector under the control of the nisin promoter; the porcine LF (PLF) cDNA fragment was PCR amplified and cloned into the *Kpn*I and *Hind*III sites of the pNZ8148 vector under the control of the nisin promoter.

### 3.2. Transfection of the Constructed Plasmids into Various Hosts Using Electroporation

To obtain various probiotic strains that could express HLF, BLF, or PLF, the constructed pNZ8148-HLF, pNZ8148-BLF, and pNZ8148-PLF were then transformed into different probiotic hosts, as shown in Table 1. Transformed cells were further selected after five rounds of chloramphenicol selection with increasing concentrations (2.5, 5, 10, 15, and 20 µg/mL). Although the same approach was employed for all competent probiotic cells, the transformed probiotic strains showed considerable differences in their response to the five rounds of chloramphenicol treatment. In other words, several transformed cells did not survive after a total of five rounds of treatment with antimicrobial agents. Nevertheless, we were able to generate 11 clones (candidates) of recombinant probiotic strains, that is, *L. delbrueckii*/HLF, *L. delbrueckii*/BLF, *P. pentosaceus*/BLF, *B. angulatum*/BLF, *B. angulatum*/PLF, *L. paraplantarum*/HLF, *L. paraplantarum*/BLF, *B. breve*/BLF, *B. catenulatum*/PLF, *L. rhamnosus*/HLF, and *L. gasseri/BLF.* Obtained cell clones were subjected to SDS-PAGE and Western blotting analysis for analyzing LF expression, as described below.

### 3.3. Expression of rLF Protein in Selected Probiotic Transformants

**Figure 2:**
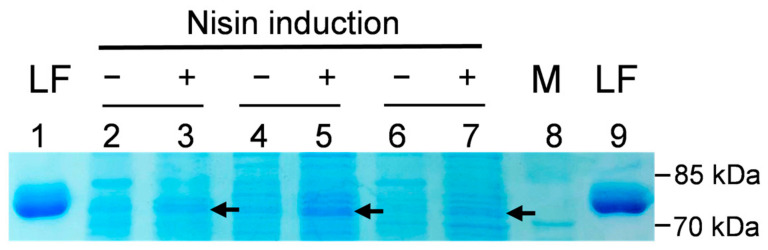
SDS-PAGE profile of recombinant lactoferrin expression. Probiotic hosts with porcine or bovine lactoferrin expression plasmids were induced (+) or non-induced (−) by lactoferrin expression using nisin for 4 h. Cells lysates were supernatant after sonication disruption and were analyzed via SDS-PAGE. LF: standard control panel, 0.32 μg purified LF; M: molecular weight marker; lane 2 to 3: *Lactobacillus delbrueckii/PLF*; lane 4 to 5: *Lactobacillus gasseri/BLF*; lane 6 to 7: *Bifidobacterium angulatum/PLF.* Arrow indicates target lactoferrin with a molecular weight of about 80 kDa.

**Figure 3:**
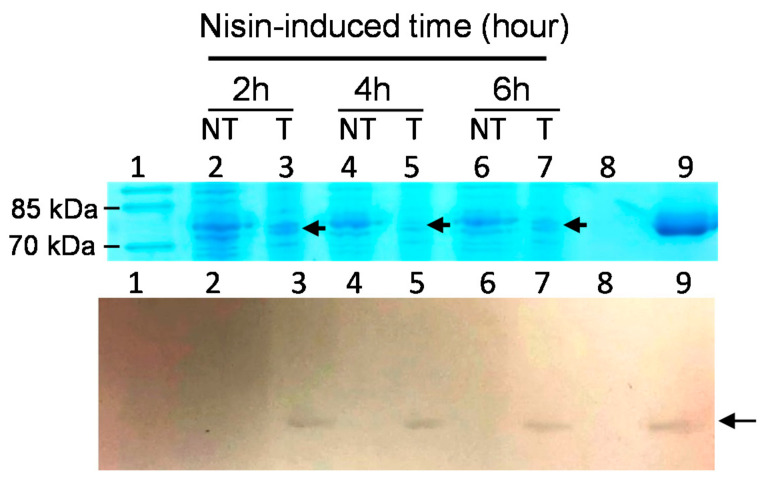
The LF expression in the transformed *Lactobacillus gasseri*/BLF or the non-transformed control. BLF expressions in *L. gasseri*/BLF or *L. gasseri* (host control) cells were all induced protein expression using 1 ng/mL nisin for 2, 4, and 6 h. The cell lysates were harvested and subjected to SDS-PAGE analysis (top panel) and Western blotting analysis (bottom panel). Lane 1: M: molecular weight marker; lane 2, 4, and 6: the non-transformed (NT) host strain; lane 3, 5, and 7: the transformed (T) *L. gasseri*/BLF. Lane 8: no sample; lane 9: 0.32 μg standard bovine lactoferrin (80 kD). Arrow indicates target lactoferrin with a molecular weight of about 80 kDa. The LF signal was detected in nisin-inducted *L. gasseri/BLF* but not in *L. gasseri* control.

**Figure 4:**
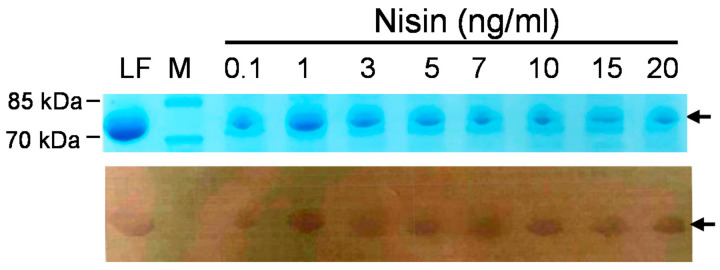
Influence of varying concentrations of nisin on recombinant lactoferrin expression. The *Lactobacillus gasseri*/BLF was induced using eight doses of nisin for 6 h, and the cell lysates were harvested and subjected to SDS-PAGE analysis (top panel) and Western blotting analysis (bottom panel). Lane LF: control well of 0.32 μg standard bovine lactoferrin (80 kDa); M: molecular weight marker; arrow indicates target lactoferrin.

SDS-PAGE profiles and Western blotting analyses were employed to evaluate and confirm the successful expression of rLF in various clones. In Figure 2, the representative results of the SDS-PAGE profiles of several rLFs expressed in probiotic hosts are shown. Here, the expression of the potential 80 kDa rLF protein was observed to be induced by the addition of nisin. Next, to confirm the expression of rLF in recombinant probiotic strains, the Western blotting analysis with rabbit anti-LF primary antibody was employed. In Figure 3, the LF expression in the transformed *Lactobacillus gasseri*/BLF or the non-transformed control is shown. The expression of rLF could be induced by 1 ng/mL of nisin, and no LF signal was detected in host bacterial strain. Then, we also invested the influence of varying concentrations of nisin on those recombinant lactoferrin expressions, and the results show that the transformed probiotic candidates showed considerable differences in their response to the nisin induction. Moreover, several transformed probiotic clones did not produce detectable LF in the preliminary analysis. The representative results of the influence of varying concentrations of nisin on rLF expression in one probiotic clone are shown (Figure 4). Here, the *L. gasseri/BLF* was induced using eight doses of nisin, and the cell lysates were harvested and subjected to SDS-PAGE analysis (top panel) and Western blotting analysis (bottom panel). The results show that the expression of rLF could be induced by 0.1 to 20 ng/mL of nisin. With the help of quantifications performed using ImageQuant 5.1 software, the densities for the various nisin effects from 0.1 to 20 ng/mL were about 20,433.95, 80,983.95, 62,775.95, 48,654.95, 58,394.95, 41,565.95, 19,439.95, and 12,775.95, respectively. Therefore, when compared to the densities of 0.1 ng/mL nisin, the LF expression ratios were found to be about 1.00, 3.96, 3.07, 2.38, 2.86, 2.03, 0.95, and 0.63 folds higher. Therefore, a nisin concentration of 1 ng/mL was found optimal for the induction of high levels of LF in the host probiotic. As partially described in the introduction section, nisin is an antimicrobial peptide (lantibiotic) that exhibits antimicrobial activity against mainly other Gram-positive bacteria by forming small pores in the cellular membrane [16]. Therefore, nisin-producing bacterial strains often display a high degree of resistance to the action of nisin, which is based upon expression and interaction of the self-protection (immunity) genes *nisI*, *nisF*, *nisE*, and *nisG* [33,34]. As for the NICE system, in general, specific hosts strains, such as *L. lactis* (NZ9000 or NZ9800), are often used. Notably, the strain NZ9800 harbors immunity genes *nisIFEG*, and, thus, it can tolerate relatively higher concentrations of nisin up to 10 mg/mL. However, the maximum induction level is often reached when 5 to 10 ng/mL nisin is administered, and a concentration-dependent induction of the interested genes will more often occur when nisin is added between 0.01 to 10 ng/mL during the log phase of bacteria [35]. In the current study, as indicated above, the highest concentration of rLFs was reached at 1 ng/mL nisin treatment, and similar rLFs levels were observed between 3 to 10 ng/mL nisin treatments. Moreover, a higher nisin concentration (15 to 20 ng/mL) range did not contribute to higher rLFs levels. Collectively, no obvious dose-dependent effect could be observed according to the current tested nisin dosages. We believe this could be due to several reasons: first, as previously explained, even though the NZ9800 is used in the NICE system, the dose-dependent induction of the genes of interest will often occur when nisin is added between 0.01 to 10 ng/mL, and the maximum induction level is often reached by using 5 to 10 ng/mL nisin; second, our host strain might not contain enough amounts of self-protection genes against nisin, and, thus, our strain could not tolerate relatively higher concentrations of inducer nisin (more than 10 ng/mL); finally, as partially explained in the introduction section, our strain might not contain enough amounts of regulatory machinery in the NICE system to express the recombinant protein in a dose-dependent manner.

Notably, Western blotting also revealed that the molecular mass of rLF was quite similar to that of the BLF standard. This implies that the rLFs produced by transformed probiotic strains likely possessed post-translational modifications similar to the purified BLF control. To support this, it has been shown that both *N*- and *O*-glycosylation, once believed to be restricted to eukaryotes, are also present in bacteria and archaea [36,37]. Furthermore, a previous report also demonstrated that the probiotic *L. rhamnosus* GG strain could secrete glycosylated Msp1 protein, indicating that post-translational modifications are present in specific probiotic strains [38]. Thus, we believe that our rLFs in cell lysates may display biological activities comparable to those of native LFs or LF standards, but this needs to be further investigated.

**Figure 5:**
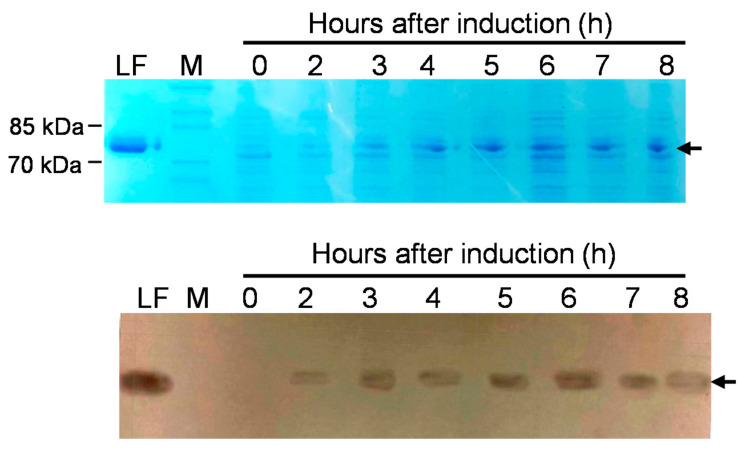
The time course of LF expression in the transformed *Lactobacillus gasseri*/BLF. BLF expression in *L. gasseri*/BLF cells was induced using 1 ng/mL nisin for 0, 2, 3, 4, 5, 6, 7, and 8 h. The cell lysates were harvested and subjected to SDS-PAGE analysis (top panel) and Western blotting analysis (bottom panel). Lane LF: control panel of 0.32 μg purified bovine lactoferrin (80 kDa); M: molecular weight marker; arrow indicates target lactoferrin with a molecular weight of about 80 kDa.

We also determined the time course of LF expression in the transformed host strains; the representative results are shown in Figure 5. Here, BLF expression in *L. gasseri*/BLF cells was induced using 1 ng/mL nisin for 0, 2, 3, 4, 5, 6, 7, and 8 h. The results show that BLF was detected and present in *L. gasseri* cultures after 2 to 8 h of induction, and the amount of LF protein expressed increased over time. For example, with the help of quantifications performed using ImageQuant 5.1, when compared to the densities of 2 h incubation, the LF expression ratios were found to be about 1, 3, 2.1, 3.3, 5.2, 3.2, and 1.9 folds higher. The molecular mass of rLF was also revealed here to be similar to that of the BLF standard. Furthermore, the highest concentration of LF protein was detected approximately 6 h after induction.

**Figure 6:**
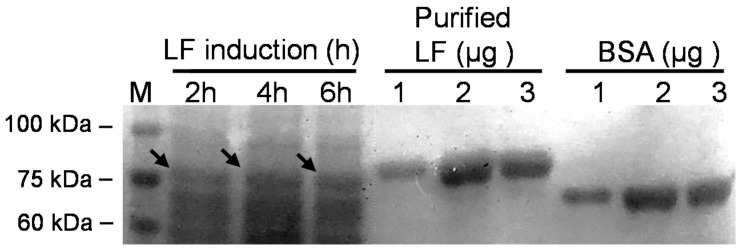
SDS-PAGE profile of recombinant BLF expressed by *Lactobacillus gasseri*. Time course of expression of recombinant BLF from *L. gasseri*/BLF cell extracts prepared from samples collected after 2, 4, and 6 h of induction using nisin. Cell lysates were harvested and subjected to SDS-PAGE analysis. Three purified LF and bovine serum albumin standards (1, 2, and 3 μg) were used to evaluate the level of recombinant LF expression in probiotic host. M: molecular weight marker; arrow indicates target lactoferrin with a molecular weight of around 80 kDa.

We also assessed the level of recombinant protein expression in *L. gasseri* by comparison with a protein standard, that is, purified LF or bovine serum albumin (Figure 6). With the help of quantifications performed using ImageQuant 5.1, we calculated the rLF concentrations based on the densities of the purified LF, and the concentrations for the expressed rLFs after 2, 4 and 6 h induction were estimated to be about 27.8 mg/L, 34.8 mg/L and 28.48 mg/L, respectively. However, this experiment was conducted to obtain some clues about the expressed rLF in a small scale and a short time culture. The concentration of rLF in the long term or a larger culture remains unclear, and we intend to dissect this in a future study.

Taken together, as shown in Table 1, we obtained 11 recombinant probiotic candidates that may produce rLFs, but the expression of rLFs was not detected in six probiotic clones, probably in a nonoptimal nisin induction fashion. For example, it is known that nisin induction is mainly based on a two-component fashion, as described in the Introduction Section, and studies also indicate that the *nisRK* genes are the only nis genes required for nisin-mediated signal transduction and *nisA* or *nisF* promoter activation in *Lactococcus lactis* [19,20]. This may explain that the expression of rLFs was not detected in six clones. Intriguingly, in the present work, the expression of rLFs was induced successfully in another five probiotic clones, namely, *L. delbrueckii*/HLF, *L. delbrueckii*/BLF, *B. angulatum*/BLF, *B. angulatum*/PLF, and *L. gasseri*/BLF. To support this, the heterologous expression of the nisin in non-*L. lactis* was reported [39], and other factors, such as galactose or lactose, can modulate the modulate the transcription of the nisin biosynthetic genes in a NisRK-independent manner [21,39]. In other words, our findings also partially support the notion that heterologous expression of the nisin in non-*L. lactis* strain is possible, but further studies are needed to elucidate the molecular machinery regarding our recombinant probiotic strains, which could express rLF or activate the nisA promoter by using only one plasmid.

### 3.4. In Vitro Antibacterial Activities of rLFs from Two Probiotic Hosts

It is known that although prokaryotic expression systems have been widely used for producing low-molecular-mass recombinant proteins, these strategies are not always successful. Therefore, we next determined whether our recombinant probiotic clones could produce functional LFs in the *L. delbrueckii* and *L. gasseri* hosts. In other words, we decided to evaluate the antibacterial activities of three recombinant clones, namely, *L. gasseri*/BLF, *L. delbrueckii/*HLF, and *L. delbrueckii*/BLF, for several reasons. Firstly, the three clones could produce relatively higher rLFs levels under various induction ways, such as induction with 0.1 to 20 ng/mL nisin, induction under the bacterial density of 0.3 to 1.5 (OD600), and induction at a temperature of 28 °C to 37 °C. Second, the two selected conventional probiotic hosts, *L. delbrueckii* and *L. gasseri* (our laboratory stock), were isolated from yogurt and human milk, respectively, revealing that the two strains originate from natural and safe sources. Finally, *L. delbrueckii* has been shown previously to display both antibacterial activities and detoxification capacity against uremic toxins in vitro [28,40]. As for *L. gasseri* (our laboratory stock), we confirmed that the milk-isolated *L. gasseri* can display anti-bacterial activities, as well as both bile salt and acid tolerance abilities in our preliminary analysis. Therefore, we speculate that the production of recombinant LFs in the two selected hosts (three recombinant probiotic clones) may enhance the beneficial effects of these probiotic strains, making use of their wider applicability.

Initially, we decided to compare the antibacterial activities between induced and non-induced *L. delbrueckii/HLF*, *L. delbrueckii/BLF*, and *L. gasseri/BLF* clones against four important food-borne pathogens by using two methods: the agar diffusion test and broth inhibition analysis. In the preliminary test, we also attempted to determine the relationship between induction time, bacterial density (probiotics), induction temperature, and antibacterial activities of probiotic clones. Finally, LF expression was induced by adding nisin at a concentration of 1 ng/mL to 24 mL probiotic culture for 16 h at 30 °C; then, the recombinant probiotic cells were harvested by centrifugation and resuspended using MRS and disrupted directly by using a sonication approach. On the other hand, to directly apply the clear supernatants prepared from recombinant probiotic strains in the fields, the fresh medium (MRS), phosphate-buffered saline, and saline were evaluated as the sonication buffer in our system. The results reveal that the antibacterial activities of probiotics could be retained simply by using MRS as a sonication buffer. As shown in Table 2, the blank control (MRS) did not show inhibition zone. In contrast, the three rLF-expressed recombinant probiotic clones displayed significantly wider inhibition zone diameters (*P* < 0.001 or *P* < 0.01) than that of the non-rLF induced probiotic strains against *S. aureus*, *En. Faecalis*, *S. enterica*, and *E. coli*. Three independent experiments were conducted, each was performed in triplicate, and similar findings were observed. Representative inhibitory zones of agar diffusion test showing the antibacterial activity of cleared supernatants prepared from induced and non-induced recombinant probiotic strains are shown in Supplementary Figure S1. Therefore, these experiments demonstrate that the recombinant HLF or BLF in *L. delbrueckii* and *L. gasseri* are soluble and functionally active proteins, and the presence of these rLFs in the probiotics further boosted their antibacterial efficacies. Intriguingly, our data also support that the MRS could be used as the sonication buffer to maintain the antibacterial activities of rLFs by using the agar diffusion test. To confirm this, we also conducted the broth microdilution analysis. For example, aliquots of the cleared supernatants from the recombinant probiotics (rLF-expressed or non-rLF-expressed) and the pathogenic bacterial solution were combined in a 96-well microplate, which was followed by incubation at 37 °C. Then, the bacterial growth (change in turbidity) was determined spectrophotometrically (wavelength of 600 nm) at different time points in a microplate reader. As expected, supernatants collected from rLF-expressed and non-rLF recombinant probiotic clones all displayed antibacterial activities against the four selected pathogens, and supernatants from the three rLF-expressed probiotic clones also displayed relatively stronger antibacterial activities than those of the non-rLF induced probiotic strains, especially at the end of the experiments (24 or 48 h time points). Therefore, these findings are in line with the results obtained in the agar diffusion test. However, we only determined the antibacterial activities of one dose of supernatants, and the antibacterial potency was quite similar among rLFs-induced and non-rLFs-induced clones in broth microdilution analysis. Then, we tried to conduct another broth-inhibition assay to visualize antibacterial activities between recombinant clones by consulting a previous report, as described below [29].

**Figure 7:**
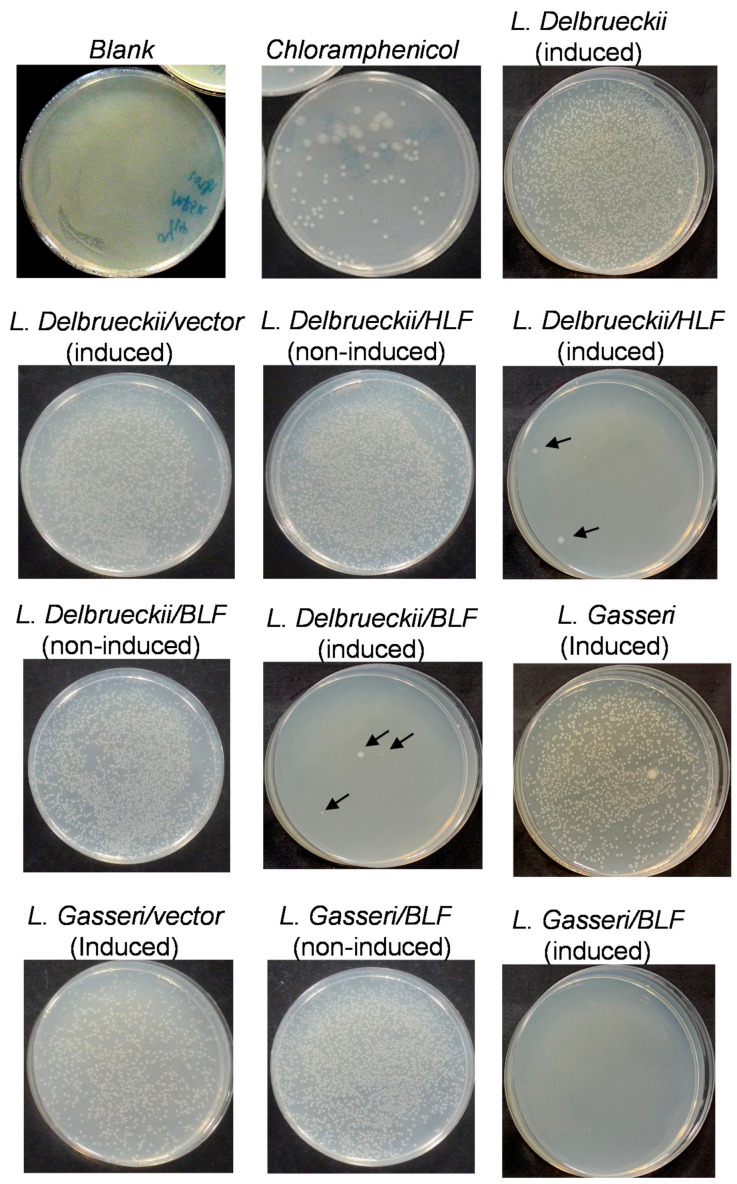
Effects of recombinant human or bovine lactoferrin cell lysates on the growth of *Staphylococcus aureus*. About 100 mL *L. delbrueckii* (host control), *L. delbrueckii/*pNZ8148 (vector control), *L. delbrueckii/HLF*, *L. delbrueckii/BLF*, *L. gasseri (host control)*, *L. gasseri/*pNZ8148 (vector control), and *L. gasseri*/BLF was induced by protein expression for 5 h using nisin. Cell pellets were harvested, washed by phosphate-buffered saline twice, and then disrupted by sonication. Supernatants (cell lysates) were then harvested by centrifugation. Supernatants (200 μL/assay) were mixed with pathogenic bacterial broth (1 × 10^4^ cfu/mL; 300 μL) in Eppendorf, and these mixers were further incubated for 24 h at 37 °C. Then, 200 μL of the mixtures was further plated onto nutrient agar (NA) plates to reveal the remaining growth of bacterial colonies. Arrows indicate the grown of individual bacterial colonies. The final concentration of 12.5 μg/mL chloramphenicol was also used as the control. The blank control presented as smear-type bacterial, revealing countless pathogenic bacterial colonies grown on the NA plates, and the induced *L. delbrueckii*/BLF, *L. delbrueckii*/HLF and *L. gasseri*/BLF almost completely blocked the growth of *S. aureus* on the NA plates.

To further evaluate and confirm the antibacterial potency between the three recombinant and conventional probiotic strains, we performed another broth-inhibition assay. In our preliminary study, we also evaluated the relationship between induction time, bacterial density (pathogens and probiotics), induction temperature, and antibacterial activities of probiotic clones. Finally, the conditions described in the Materials and Methods Section were found to easily display the antibacterial activities between probiotics by enumerating or visualization of the growth of the pathogenic bacteria on NA plates. For example, in Figure 7, the effects of rLFs cell lysates on the growth of *S. aureus* are shown. Here, the blank control (Tris and MRS) presented as smear-type bacterial cultures (pathogens), revealing countless pathogenic bacterial colonies grown on the NA plates. As for the positive control, chloramphenicol inhibited the growth of *S. aureus* strain considerably, but it did not block it completely. When supernatants prepared from conventional probiotic strains were applied, the two conventional probiotic strains also showed similar efficacy against *S. aureus*, and quite a few pathogenic bacterial colonies still survived on the respective NA plates. For example, according to the counts of bacterial colonies survived on the NA plates, both *L. delbrueckii* and *L. gasseri* displayed relatively minor activity against the growth of *S. aureus*. Furthermore, when supernatants prepared from *L. delbrueckii/*pNZ8148 and *L. gasseri/*pNZ8148 (the vector control strains) were applied, the two control strains showed similar efficacy against *S. aureus*, but quite a few pathogenic bacterial colonies still survived on the respective NA plates. Moreover, *L. delbrueckii/*pNZ8148 and *L. gasseri/*pNZ8148 also displayed similar antibacterial activities to those of the host control strains (*L. delbrueckii* and *L. gasseri*). In comparison, the same doses of supernatants prepared from induced *L. delbrueckii/BLF*, *L. gasseri/BLF*, and *L. delbrueckii/HLF*, these almost completely blocked the growth of the *S. aureus* strain, revealing no bacterial colonies (or up to 3 colonies) on the NA plates. Additionally, the induced *L. delbrueckii/BLF*, *L. gasseri/BLF*, and *L. gasseri/BLF* also displayed stronger antibacterial activities than the non-induced strains. Furthermore, the effects of rLFs cell lysates on the growth of four pathogenic strains are shown in Supplementary Figures S2–S4, and similar findings have been previously observed.

Collectively, the findings of the agar diffusion test and broth dilution analysis all indicate that the expression of rLFs in *L. delbrueckii*/HLF, *L. delbrueckii*/BLF, and *L. gasseri*/BLF clones considerately improves their antibacterial efficacies. Moreover, these results may also support the notion that the combination of LF with specific probiotic strains which already display antibacterial activities could contribute to synergistic or additive antibacterial potency, as described in our previous report [23,24]. Since the antibacterial potency between purified BLF or HLF (from milk) and our recombinant LFs (from probiotics) could be different, we will further examine the antibacterial activity by adding purified LF to lysates of *L. delbrueckii*/pNZ8148 and *L. gasseri*/pNZ8148 (the vector control strains) in our next study. Nevertheless, our data provide a potential and useful strategy for the production of functionally recombinant LFs in functional hosts for applications in the field. Notably, as partially explained in the Introduction Section, in general, in the nisin-inducible expression system, a specific host strain that harbors nisK and nisR or, alternatively, a transferable dual-plasmid inducible gene expression system should be used to express interested proteins [19,20], and in the present work, we successfully expressed rLFs in specific hosts using only one plasmid.

In support of our findings, a recent study showed that the culture supernatant of *L. delbrueckii* (BCRC 14008), which is the same bacterial strain used in the current study, could display antibacterial activities against the growth of *Klebsiella pneumoniae* (BCRC 10694) but could not inhibit the growth of *Gardnerella vaginalis* (BCRC 17040), using an agar diffusion test [28]. However, in the report, it was not further evaluated whether the antibacterial activities of *L. delbrueckii* could be attributed to the production of organic acids or bacteriocin. Notably, in the current study, we assessed the antibacterial activities of cleared supernatants prepared from disrupted probiotic cells, but not from culture supernatants, and the ruptured cell lysates displayed antibacterial potency against selected food-borne pathogens. Thus, we provide new support that *L. delbrueckii* may also secrete bacteriocin, but this needs to be further investigated. On the other hand, the previous study also indicated that *L. delbrueckii* is not active against the growth of *G. vaginalis*, an important etiology of bacterial vaginosis in humans [28]. Thus, it will be interesting to test the antibacterial potency of our recombinant *L. delbrueckii/HLF* and *L. delbrueckii/BLF* clones against the growth of *G. vaginalis* in our next study. Furthermore, as for the beneficial effects of conventional *L. delbrueckii*, this strain has also been shown to reduce uremic toxin levels, mainly those of indoxyl sulfate, in vitro [40]. Therefore, we believe that the *L. delbrueckii*/BLF and *L. delbrueckii/*HLF strains engineered in our study may possess the beneficial characteristics of both conventional strains and biological activities of LFs, and it will be intriguing to observe whether the detoxification efficiency of recombinant *L. delbrueckii*/BLF and *L. delbrueckii/*HLF against uremic toxins can be confirmed in a future study.

Some previous studies have shown that the combination of probiotics with LFs can contribute to stronger anti-bacterial activities. For example, a combination of *L. acidophilus* LMG S-29159 and *L. rhamnosus* SD5675 with BLF (Respecta^®^ complex) resulted in significant inhibition of *G. vaginalis* adherence to HeLa cells [41]. Additionally, BLF has been reported to enhance *L. acidophilus* LMG S-29159 and *L. rhamnosus* SD5675 biofilm formation in a dose-dependent manner [42]. However, although it is convenient to directly combine natural LF with natural probiotic together for use in various applications, the purified mammalian LFs are quite expensive. Thus, since our system provides the conventional probiotic strains in combination with rBLF and rHLF together (LF expression probiotic clones), it might be a good alternative to the aforementioned strategy. Furthermore, previous studies have shown that natural LFs also possess anti-cancer, anti-inflammatory, or immune-regulatory activities [1,2,3], and, thus, our functional rLFs present in cell lysates here may also display these functions of natural LFs, and we will further dissect these issues in our next study.

Our findings also reveal that the milk-isolated bacterial strain, *L. gasseri*, may secrete bacteriocin to contribute to antibacterial activities. This finding is important and could partially support previous findings on the roles of probiotic members in milk in controlling bacterial mastitis [43,44,45]. Moreover, in the present study, the supernatants of *L. gasseri/BLF* had much stronger antibacterial effects than those of the conventional *L. gasseri* strain against selected pathogens, supporting the notion that the combination of LF and the milk-isolated probiotic strain may contribute to synergistic or additive antibacterial potency, as previously reported [23,24].

It is known that fast and high-level expression of recombinant proteins in bacterial hosts often leads to an accumulation of inclusion bodies (insoluble aggregates) of the target protein. These inclusion bodies often require extensive processing to recover and produce bioactive proteins [46,47]. In the current study, we provide evidence that rLFs present in cell lysates of recombinant probiotics are soluble and functionally active proteins by using two antibacterial analyses, and this can be easily used in various other applications. Notably, 5 to 7 h was found to be sufficient for functional LF production by the three transformed probiotic clones. We have previously shown that the combination of purified BLF (from milk) and specific probiotics can lead to synergistic antibacterial activity in vitro [23,24], and in this study, our results reveal that the strategy of a combination of recombinant LFs and specific probiotics can be easily obtained by our recombinant *L. delbrueckii*/BLF and *L. delbrueckii*/HLF and *L. gasseri*/BLF. Thus, these may represent promising preventative and therapeutic anti-bacterial agents in the relevant fields. However, it should be indicated that the use of probiotics may have its downsides. For example, strains of lactobacilli producing bacteriocins and other antimicrobial substances could display “antibiotic-like” effects for short duration, and this may cause a dysbiotic gut microbiota, leading to other health problems [48]. For instance, a previous study reports that in patients with predicted severe acute pancreatitis, probiotic prophylaxis did not reduce the risk of infectious complications but was associated with an increased risk of mortality [49]. Additionally, evidences also support that specific probiotics or bacteriocins could also affect normal gut microbiota and could play different roles in weight gain or loss in human and animals [50]. Moreover, commensal bacteria, including lactic acid bacteria, may also act as reservoirs of antibiotic resistance genes similar to those found in human or animal pathogens [51]. On the other hand, an early report indicates that there may be a relation between virulence of bacteria and resistance to LF, and several bacterial strains have been observed to be LF-resistant bacteria [52]. However, in general, LF-related proteins or peptides have been shown to be sensitive to most of the tested bacterial strains, including antibiotic-resistant bacteria [24,53,54].

Collectively, this study aims to promote the constructed recombinant *Lactobacilli* as a promising producer of LF and to further utilize the recombinant microorganisms using the disrupted probiotic lysates. These cell lysates do not carry transferable antibiotic resistance that could be transferred to commensal or pathogenic bacteria. Furthermore, to support the safety issues about the host bacterial strains, the selected *L. delbrueckii* here is originated from commercial yoghurt, and *L. gasseri* is isolated from human milk. The later strain was sensitive to oxacillin, ampicillin, cephalothin, amoxicillin, erythromycin, clindamycin, and oxytetracycline in our preliminary test.

### 3.5. Effects of Recombinant Human or Bovine Lactoferrin on the Growth of Recombinant L. gasseri/BLF, L. delbrueckii/HLF, and L. delbrueckii/BLF

**Figure 8:**
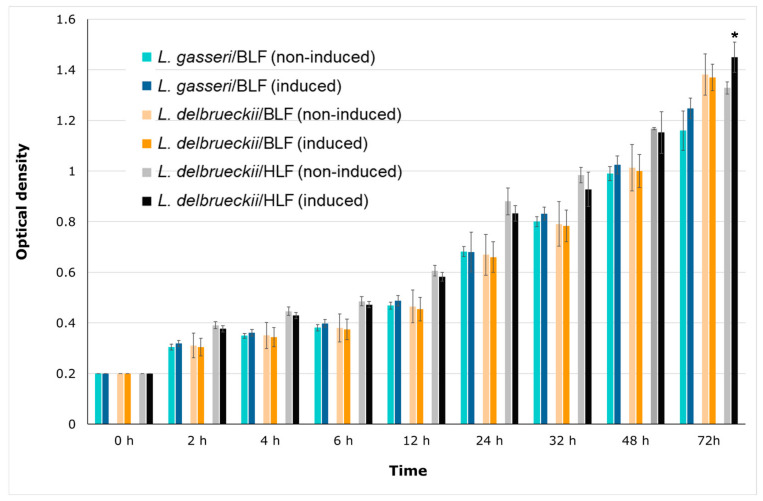
Effects of recombinant human or bovine lactoferrin on the growth of three recombinants: *L. gasseri*/BLF, *L. delbrueckii/*HLF, and *L. delbrueckii*/BLF. The recombinant probiotic strains were grown in deMan–Rogosa–Sharpe (MRS) broth to an optimal optical density at 600 nm (OD_600_) of 0.2. Then, theses bacteria were cultured in MRS medium with and without nisin-induced expression of recombinant human or bovine lactoferrin. The growth responses of each bacterium were measured by determining the optical density at different time intervals at room temperature (approximately 23–25 °C). The results are expressed as mean ± standard deviation and variations in growth curves of probiotic bacteria with and without nisin induction. * Significant differences in the probiotic growth with and without nisin induction (*P* < 0.05).

The above results support the notion that the three recombinant probiotic candidates display stronger antibacterial activities than those of conventional strains and non-nisin-induced controls, indicating that these recombinant probiotics could produce soluble and functional recombinant lactoferrin. As explained elsewhere in this study, probiotic strains that display enhanced growth rates in the presence of BLF, or those that can withstand the antibacterial activity of BLF, were used as host-friendly bioreactors to produce recombinant rLFs. Therefore, we further determined the growth rate between induced and non-induced *L. delbrueckii/HLF*, *L. delbrueckii/BLF L.*, and *L. gasseri/BLF* clones cultured at room temperature (approximately 23–25 °C) or 37 °C. In Figure 8, nisin-induced expression of rBLF did not significantly affect the growth rate of *L. gasseri*/BLF and *L. delbrueckii*/BLF. Moreover, nisin-induced expression of rHLF slightly and significantly (*P* < 0.05) elevated the growth rate of *L. delbrueckii*/HLF only at 72 h, indicating rHLF could play a role in promoting the growth rate of *L. delbrueckii/*HLF. In our previous studies [4,55], we only determined the prebiotic effects of purified BLF but not the HLF on the growth of a series of probiotic strains. Therefore, the current study has provided new clues that HLF could also slightly promote the growth on specific probiotic strains. The same experiment was also conducted at 37 °C, and similar findings were found. Collectively, these data show that rLFs do not markedly affect the growth rate of *L. delbrueckii/HLF*, *L. delbrueckii/BLF*, and *L. gasseri/BLF*. The different effects of purified LF or our rLFs on the growth of probiotics may be partially explained by inconsistent assay strategies and different LF purities or by the fact that concentrations were modified when compared to those of our previous report. For example, in our previous report, purified BLF was added to the medium directly, and then we evaluated the effects of BLF on the growth of tested probiotics [4]. However, in the current study, the rLFs are expressed within the bacterial cells, and these proteins are not released to the medium by the hosts. Nevertheless, the findings in Figure 8 support the notion that nisin addition or rLFs expression does not affect the growth rate of *L. delbrueckii*/HLF, *L. delbrueckii*/BLF, and *L. gasseri*/BLF, indicating that these recombinant probiotics are still LF resistant. On the other hand, the stability or concentration of LF in long-term culture remains unclear, and we intend to dissect that in a future study.

## 4. Conclusions

In this study, by taking the GMO issues into account, we focused on using disrupted probiotic lysates containing functional rLFs for further applications. Since the sonication can disrupt both the bacteria and the plasmids within them, the use of cell lysates instead of GMO probiotics might be better accepted by the public. Here, our results demonstrate that the cleared supernatants prepared from disrupted cell lysates contain soluble and functional LFs, and we believe that these engineered probiotic clones may provide both the beneficial characteristics of lactic acid bacteria and the biological activity of LF. Alternatively, a combinational approach of alive conventional probiotic strains with cleared supernatants prepared from recombinant probiotic cells could be used (providing the rLFs), as all of the probiotic hosts selected were able to resist the antibacterial activities of LFs. Nevertheless, our data provide a simple strategy for the production of recombinant LFs in LF-resistant hosts for applications in the field.

## Figures and Tables

**Table 1:** Selection of probiotic strains with transformed vectors as the stable clones.

	Plasmid		
Probiotic	pNZ8148 /HLF	pNZ8148 /BLF	pNZ8148 /PLF
*Lactobacillus delbrueckii*	+ ^1^	+	− ^2^
*Pediococcus pentosaceus*	−	+	−
*Bifidobacterium angulatum*	−	+	+
*Lactobacillus paraplantarum*	+	+	−
*Bifidobacterium breve*	−	+	−
*Bifidobacterium catenulatum*	−	−	+
*Lactobacillus rhamnosus*	+	−	−
*Lactobacillus gasseri*, strain A (Laboratory stock)	−	+	−

^1^ Indicates that the transformed bacterial clone was further selected and survived after five rounds of chloramphenicol selection with increasing concentrations (2.5, 5, 10, 15, and 20 µg/mL). ^2^ indicates the transformed bacterial clone did not survive after five rounds of chloramphenicol selection.

**Table 2:** Average inhibitory zone against food-borne pathogens of recombinant probiotic strains. Agar diffusion test showing the antibacterial activity of cleared supernatants prepared from induced and non-induced recombinant probiotic strains.

	Inhibitory Zone Diameter (mm) ^1^			
*Lactobacillus* Strains	*S. aureus*	*En. faecalis*	*S. enterica*	*E. coli*
*L. delbrueckii/HLF* (induced) ^2^	14.8 ± 0.7 ***	14.8 ± 0.5 ***	15.8 ± 1.4 ***	15.0 ± 2.0 **
*L. delbrueckii/HLF* (non-induced) ^2^	12.7 ± 0.9	12.5 ± 1.0	12.9 ± 0.7	12.4 ± 0.9
*L. delbrueckii/BLF* (induced) ^2^	14.5 ± 1.3 ***	15.3 ± 1.1 ***	15.3 ± 1.7 **	14.9 ± 1.3 **
*L. delbrueckii/BLF* (non-induced) ^2^	12.5 ± 0.9	12.8 ± 1.1	12.7 ± 0.9	13.0 ± 0.9
*L. gasseri/BLF* (induced) ^2^	14.8 ± 0.3 ***	15 ±0.9 ***	14.9 ± 0.9 ***	15.1 ± 0.9 ***
*L. gasseri/BLF* (non-induced) ^2^	12.4 ± 0.3	13.1 ± 0.4	13.1 ± 0.4	13.4 ± 0.8
Blank control	10 ± 0	10 ± 0	10 ± 0	10 ± 0

^1^ Data represent the mean (± standard deviation, SD) of three independent experiments, each performed in triplicate. ^2^ Induced or non-induced: this strain was induced or non-induced to express recombinant lactoferrin; HLF: human lactoferrin; BLF: bovine lactoferrin. ** *P* < 0.01, *** *P* < 0.001: indicates a significant difference with the non-induced samples.

## Data Availability

Data is contained within the article or supplementary material.

## Supplementary Materials

The following are available online at https://www.mdpi.com/2079-6382/10/2/120/s1, Figure S1: Representative inhibitory zones of agar diffusion test showing the antibacterial activity of cleared supernatants prepared from induced (+) and non-induced (-) recombinant probiotic strains. See materials and methods section for the experiment design. Figure S2: Effects of recombinant human lactoferrin cell lysates on the growth of four pathogenic bacterial strains. Figure S3: Effects of recombinant bovine lactoferrin cell lysates on the growth of four pathogenic bacterial strains. Figure S4: Effects of recombinant bovine lactoferrin cell lysates on the growth of four pathogenic bacterial strains.
