# Supplementary material for: A Single Plasmid of Nisin-Controlled Bovine and Human Lactoferrin Expressing Elevated Antibacterial Activity of Lactoferrin-Resistant Probiotic Strains
Source: Antibiotics (Basel). 2021 Jan 27;10(2):120. doi: 10.3390/antibiotics10020120 (PMC7911973; doi:10.3390/antibiotics10020120)
Supplement: Supplementary file 1 [file antibiotics-10-00120-s001.pdf]

antibiotics

MDPI

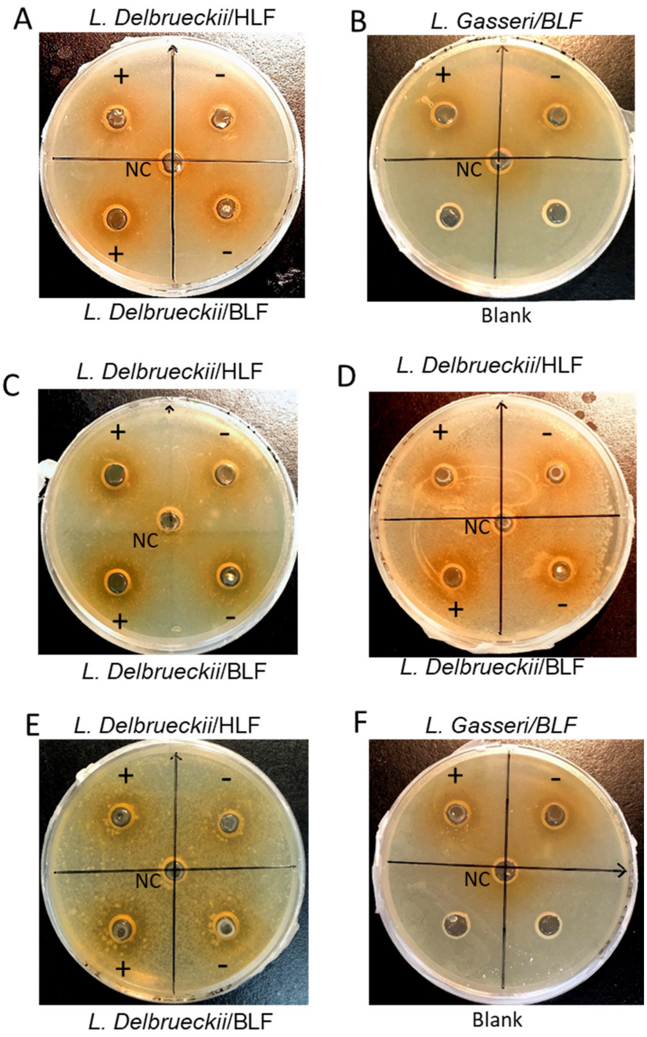

Figure S1. Representative inhibitory zones of agar diffusion test showing the antibacterial activity of cleared supernatants prepared from induced (+) and non-induced (-) recombinant probiotic strains. See materials and methods section for the experiment design. The tested pathogens are (A): *Escherichia coli;* (B)*Salmonella*; (C) *Enterococcus faecalis*; (D)*Salmonella enterica*; (E)*Staphylococcus aureus*; (F)*Staphylococcus aureus*; NC: negative control (MRS medium).

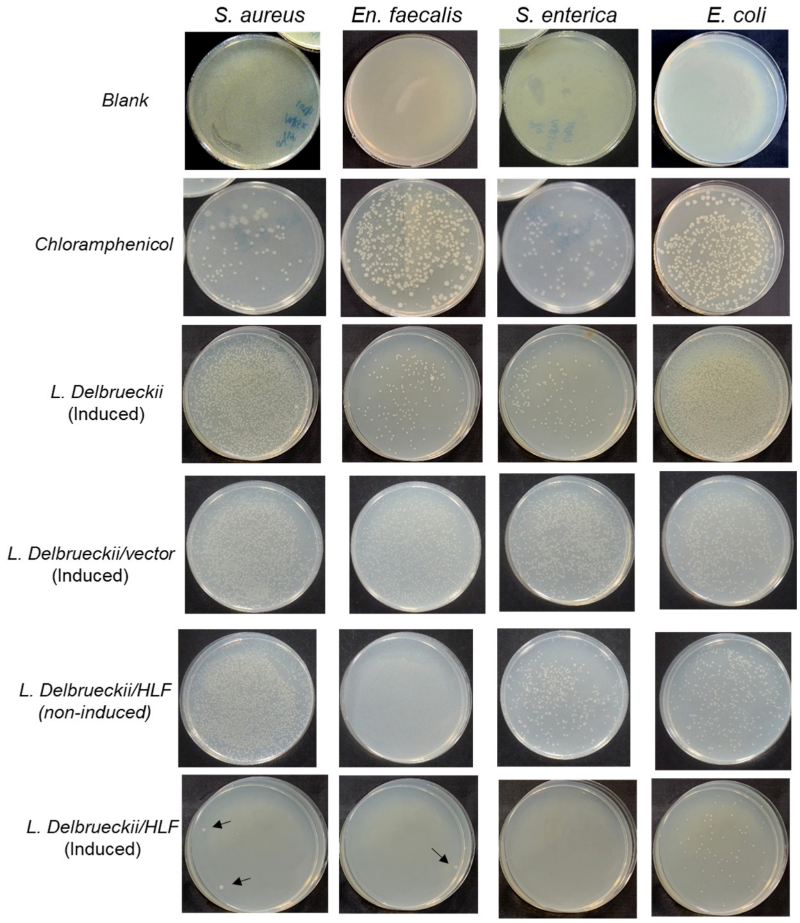

Figure S2. Effects of recombinant human lactoferrin cell lysates on the growth of four pathogenic bacterial strains. About 100 ml L. Delbrueckii (host control), L. Delbrueckii/pNZ8148 (vector control), L. Delbrueckii/HLF were induced protein expression for 5 hours using nisin. Cell pellets were harvested, washed by PBS twice, and then disrupted by sonication. Supernatants (cell lysates) were then harvested by centrifugation. Supernatants (200 μL/assay) was mixed with pathogenic bacterial broth (1×10^4 cfu/mL; 300 μL) in eppendorf, and these mixers were further incubated for 24 h at 37 °C. Then, 200 μL of mixtures were further plated onto NA plates to reveal the remaining growth of bacterial colonies. The blank control presented as smear-type bacterial cultures, revealing countless pathogenic bacterial colonies grown on the NA plates. The final concentration of 12.5 μg/mL chloramphenicol were also used as the control. Arrows indicated the grown of individual bacterial colonies, and the induced L. Delbrueckii/HLF almost completely blocked the growth of *S. aureus, En. Faecalis*, and *S. enterica.*

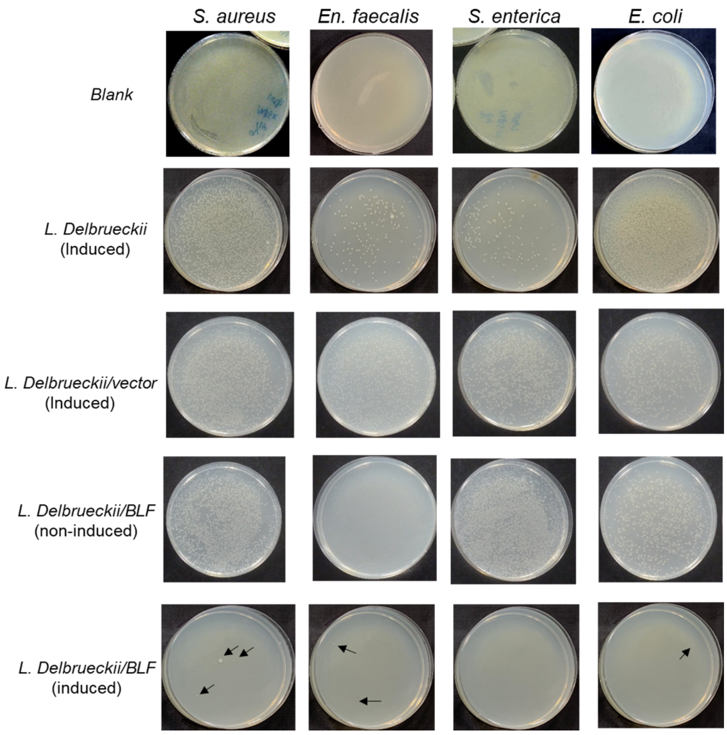

Figure S3. Effects of recombinant bovine lactoferrin cell lysates on the growth of four pathogenic bacterial strains. About 100 ml *L. Delbrueckii* (host control), *L. Delbrueckii*/pNZ8148 (vector control), *L. Delbrueckii/BLF* were induced protein expression for 5 hours using nisin. Cell pellets were harvested, washed by PBS twice, and then disrupted by sonication. Supernatants (cell lysates) were then harvested by centrifugation. Supernatants (200 μL/assay) was mixed with pathogenic bacterial broth (1×10^4 cfu/ml; 300 μL) in eppendorf, and these mixers were further incubated for 24 hours at 37°C. Then, 200 μL of mixtures were further plated onto NA plates to reveal the remaining growth of bacterial colonies. The blank control presented as smear-type bacterial cultures, revealing countless pathogenic bacterial colonies grown on the NA plates. Arrows indicated the grown of individual bacterial colonies, and the induced *L. Delbrueckii*/BLF almost completely blocked the growth of *S. aureus, En. Faecalis,S. enterica,* and *E. coli.*

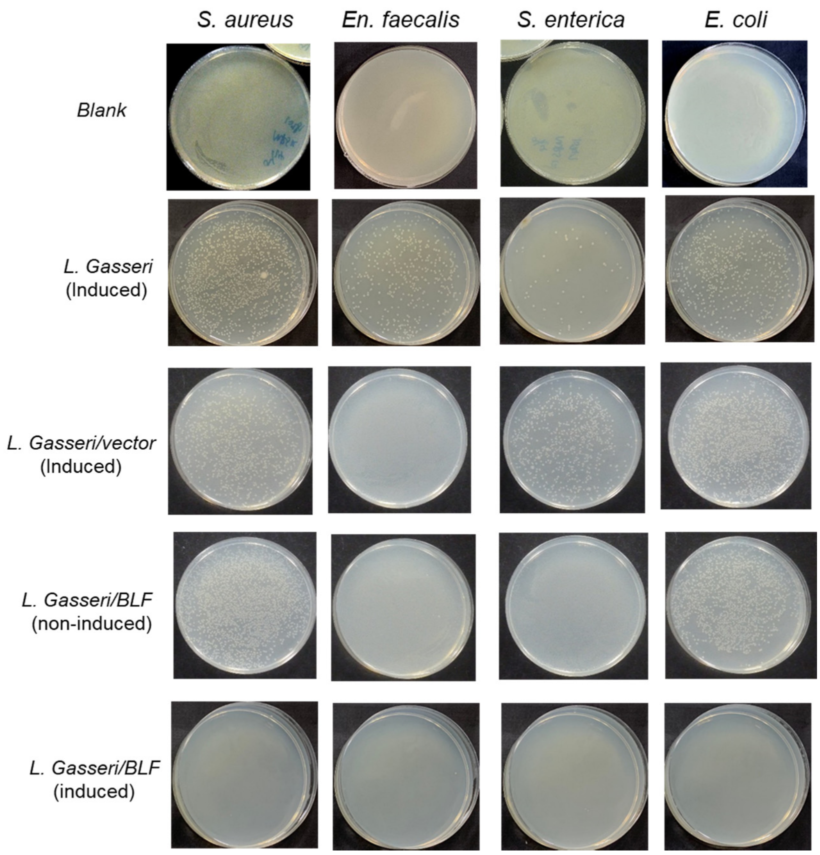

Figure S4. Effects of recombinant bovine lactoferrin cell lysates on the growth of four pathogenic bacterial strains. About 100 mL *L.Gasseri* (host control), *L. Gasseri*/pNZ8148 (vector control), *L. gasseri/BLF* were induced protein expression for 5 hours using nisin. Cell pellets were harvested, washed by PBS twice, and then disrupted by sonication. Supernatants (cell lysates) were then harvested by centrifugation. Supernatants (200 μL/assay) was mixed with pathogenic bacterial broth (1 × 10^4 cfu/mL; 300 μL) in eppendorf, and these mixers were further incubated for 24 h at 37 °C. Then, 200 μL of mixtures were further plated onto NA plates to reveal the remaining growth of bacterial colonies. The blank control presented as smear-type bacterial cultures, revealing countless pathogenic bacterial colonies grown on the NA plates. Arrows indicated the grown of individual bacterial colonies, and the induced *L. gasseri*/BLF almost completely blocked the growth of *S. aureus*, *En. Faecalis*, *S. enterica*, and *E. coli.*
